# Is the HERV-K HML-2 Xq21.33, an endogenous retrovirus mutated by gene conversion of chromosome X in a subset of African populations, associated with human breast cancer?

**DOI:** 10.1186/s13027-020-00284-w

**Published:** 2020-03-07

**Authors:** Mark H. Kaplan, Rafael Contreras-Galindo, Evelyn Jiagge, Sofia D. Merajver, Lisa Newman, Galya Bigman, Michael H. Dosik, Ganesh S. Palapattu, Javed Siddiqui, Arul M. Chinnaiyan, Sally Adebamowo, Clement Adebamowo

**Affiliations:** 1 Department of Internal Medicine, University of Michigan, Ann Arbor, MI 48109 USA; 2 Hormel Institute, University of Minnesota, Mayo Clinic, Austin, MN 55912 USA; 3 Henry Ford Cancer Institute, Henry Ford Health System, Detroit, Mi USA; 4 Rogel Cancer Center, University of Michigan, Ann Arbor, MI 48109 USA; 5 Weill Cornell Medicine, New York, NY 10021 USA; 6 Institute of Human Virology, University of Maryland School of Medicine, Baltimore, MD 21201 USA; 7 Department of Internal Medicine, Renaissance School of Medicine at Stony Brook Medical, Stony Brook, NY 11794 USA; 8 Department of Urology, University of Michigan, Ann Arbor, MI 48109 USA; 9 Department of Pathology, University of Michigan, Ann Arbor, MI 48109 USA; 10 Greenebaum Comprehensive Cancer Center, University of Maryland School of Medicine, Baltimore, MD 21201 USA

**Keywords:** HERV-K, HERV-K HML-2, Xq21.33, Endogenous viruses, Gene conversion, Breast cancer

## Abstract

The human endogenous retroviruses HERV-K HML-2 have been considered a possible cause of human breast cancer (BrC). A HERV-K HML-2 fully intact provirus Xq21.33 was recently identified in some West African people. We used PCR technology to search for the Xq21.33 provirus in DNA from Nigerian women with BrC and controls. to see if Xq21.33 plays any role in predisposing to BrC. This provirus was detected in 27 of 216 (12.5%) women with BrC and in 22 of 219 (10.0%) controls. These results were not statistically significant. The prevalence of provirus in premenopausal control women 44 years or younger [18/157 (11.46%)} vs women with BrC [12/117 (10.26%)] showed no statistical difference. The prevalence of virus in postmenopausal control women > 45 yrs. was 7.4% (4/54) vs 15.31% (15/98) in postmenopausal women with BrC. These changes were not statistically significant at <.05, but the actual *p* value of <.0.079, suggests that Xq21.33 might play some role in predisposing to BrC in postmenopausal women. Provirus was present in Ghanaian women (6/87), in 1/6 Pygmy populations and in African American men (4/45) and women (6/68), but not in any Caucasian women (0/109). Two BrC cell lines (HCC 70 and DT22) from African American women had Xq21.33. E*nv* regions of the virus which differed by 2–3 SNPs did not alter the protein sequence of the virus. SNP at 5730 and 8529 were seen in all persons with provirus, while 54% had an additional SNP at 7596.Two Nigerian women and 2 Ghanaian women had additional unusual SNPs. Homozygosity was seen in (5/27) BrC and (2/22) control women. The genetic variation and homozygosity patterns suggested that there was gene conversion of this X chromosome associated virus. The suggestive finding in this preliminary data of possible increased prevalence of Xq21.33 provirus in post-menopausal Nigerian women with BrC should be clarified by a more statistically powered study sample to see if postmenopausal African and/or African American women carriers of Xq21.33 might show increased risk of BrC. The implication of finding such a link would be the development of antiretroviral drugs that might aid in preventing BrC in Xq21.33+ women.

## Background

Much is lacking in our understanding of the causes of human breast cancer (BrC). There is some evidence that a mouse mammary tumor virus (MMTV) may be associated with, and be a cause of some human BrC. Such a virus is often called human mammary tumor virus (HMTV). The biology of MMTV has been summarized by Dudley et al. [1] with much speculation about how MMTV might relate to the development of BrC in humans. Numerous studies over the last 40 years have been extensively reviewed by Amarante et al. [2]. To date, MMTV does not appear to be causative of human BrC. As an animal model, it is clear that, at least in the mouse, this virus depends on breast feeding for transmission. It produces cancer later in the age of the mouse, and it does not carry any oncogenes, but depends on its insertion site near growth factors in the mouse genome to be able produce BrC [1, 2].

The human endogenous retroviruses HERV-K HML-2 have also been implicated as having some role in the pathogenesis of BrC as discussed below. These retroviruses began to enter the evolving primate genome over 35,000,000 years ago (MYA) through infection, integrating in the genome of oocyte or spermatocyte cells. After fertilization, offspring of these infected animals now contained a provirus in every cell. If animals survived this infection they could pass this provirus from generation to generation [3]. With continued evolution, subsequent infections occurred in such animals from uncertain sources, perhaps from other locally infected simians, rodents, and later in evolution from man. These newly acquired viruses could again enter into an ovary or spermatocyte and reinfect the genome in a different genomic location [4–14]. Many of these viruses entered the human genome “after the hominid-chimpanzee split 6 million years ago (MYA)” [6, 7]. These proviruses are divided into type 1, which have a 292 bp deletion in the *env* gene, and type 2, which have a more complete *env* gene. With time, most of these proviruses developed lethal mutations and indels which prevented any subsequent activation of infectious virus in the host. In addition, as these viruses had identical long terminal repeats (LTRs) on both the 5′ and 3′ side of the virus at the time of integration, through homologous recombination during cell replication some of these integrated viruses deleted all internal genes but solo LTRs which have remained in many thousand genomic locations in the hominid genome.

Much work has been done recently to fully detail the total number of HERV-K HML-2 proviruses in the human genome. Most of these proviruses are in all humans, but some have arisen in limited areas of the world and in limited populations, occurring at different times in evolution with variable spread throughout the world. Proviruses are now classified by their location in the chromosomal arm of the human genome. It is estimated that 117 of these proviruses remain in the human genome with several thousands of solo LTRs [6, 7, 9, 14–20]. Recently some 80 to 90 proviruses, called K222 were discovered which are mostly in the pericentromere of chromosomes 13, 14 and 15 [21]. In addition up to a few 1000 HERV-K HML-2 proviruses were found, called K111 [22–24], which exist mostly in the pericentromeric area primarily in chromosome 21 and 22. While the chromosomal proviruses entered the genome through either exogenous infection or endogenous reinfection, K111 and K222 probably entered the pericentromere and peripheral centromeric area first by infection. Later they appear to have spread extensively and rapidly in modern man by homologous recombination throughout these areas of repetitive elements.

Almost all of the currently known HERV-K HML-2 proviruses do not have fully functional viral genes. However 3 proviruses, K113 (19p13.11), K115 (8p23.1) and a recently discovered provirus Xq21.33 appear to have intact *gag*, *prot*, *pol* and *env* genes [25–32]. K113 and K115 are not in all humans, and their distribution varies across the world. K113 is felt to have arisen in Africa, but is present in disparate populations. In Poland, as an example, the frequency of K113 is 11.8 and for K115 it is 7.9% [32]. In a cohort of women with BrC K113 was in 16.7% and K115 was in 4.9% [27] of women tested. In Sub-Saharan Africa K113 was found in 21.8% and K115 in 34.1% [28] of patients with BrC. In the 1000 Genomes Project (1KGP) database K113 was found in 27% of all samples and 52% of samples of African origin [31]. K113 is estimated to have arisen in the human genome about 0.8MYA while K115 arose about 1.0MYA [28]. K113 appears to be able to make intact viral particles when inserted in a baculovirus expression system and is expressed in insect cells, but these lacked functional protease or reverse transcriptase activity [26]. Similarly, when K113 was cloned into a modified pBluescript vector, while some viral proteins could be made, there was “inefficient particle formation, impaired synthesis of the Gag-Pro-Po precursor and a lack of envelope protein incorporation” felt to be “key factors in HER-K113 loss of replication capacity” [25].

Xq21.33, was recently discovered by mining whole genome sequencing data (WGS) from the 1KGP. This provirus had a sample wide allele frequency of 0.0157 (frequency of 0.026 to 0.069 in African populations) mostly in Nigerian, Gambian, Kenyan and Pygmy populations [31]. This virus was also detected at low frequency in African Americans from the southeastern United States and persons living in the Caribbean, but not in other African persons living in other parts of the world. This virus has been estimated to have entered the human genome about 0.67 to 1.3MYA [31]. It appears to be fully intact and could potentially be activated. The relative rarity, as well as the potential to replicate, made this virus very intriguing to study to see if carriers of Xq21.33 in African populations might be more likely to develop BrC than non-carriers, and to understand the evolution of this virus’ sequence in different areas of Africa, and in African populations who were forced out of Africa to America via the slave trade.

## Methods

### Study subjects, cell lines and DNA samples

Women with BrC provided a consent to participate in a large study of BrC being carried out in Nigeria. These women donated peripheral blood mononuclear cells (PBL) for DNA analysis for genome wide association studies (GWAS) as well as for whole genome sequencing studies. Age matched controls, ages 19–82 years, in neighboring clinics were also asked to participate in this study. The study was conducted at the Institute of Human Virology Nigeria, an affiliate of the Institute of Human Virology, University of Maryland, Baltimore. DNA from PBL from African American men with prostate cancer was obtained from a large study of prostate cancer carried out at the University of Michigan.

DNA was extracted from saliva samples obtained during clinic visits from women in Ghana or from African American women in the United States with a history of BrC .

Additional germline DNA from women with BrC in the United States and patients with HIV and other disorders was obtained from an earlier study of K111 virus in health and disease [24].

BrC cell lines were obtained from a laboratory at the University of Michigan that had all cell lines fingerprinted before use.

Cell lines from African American patients were obtained by EJ and SM including breast cancer cell line HCC70 (ATCC® CRL-2315™) [triple negative primary ductal carcinoma], MDA MB157 (ATCC® HTB-24™) from medullary carcinoma, MDA B 468 from a pleural effusion, HCC1806 (ATCC® CRL-2335™) primary acantholytic squamous cell carcinoma, SUM 149PT from a xenograft of a transplanted primary explant of a human invasive infiltrating duct carcinoma and ZR7530 (ATCC® CRL-1504™) from metastatic ductal carcinoma. Cell lines were grown in media suggested by the company providing such lines. Other cell lines were established in our laboratory from tumor biopsies obtained from left over pathology samples. This material was obtained with informed consent with the donor remaining unknown but the pathology of the tumor known. This protocol which allowed cultivation of cells was approved by the University of Michigan Investigation Review Board (IRB). This included cell lines 09–1521, 12–1697, 12–135, 13–1357, 14–130, 15–107. Cell lines K151 and NSUH1 were established at North Shore University Hospital, (NSUH) Manhasset N.Y., from pleural effusions cells obtained with informed consent in a protocol approved by the NSUH IRB. These cells were grown in RPMI 1640 with penicillin and streptomycin and 10% heat inactivated calf serum.

DNA was extracted from PBL and cell lines using the DNeasy blood and tissue kit™ and/or the Qiagen Gentra Purgene® assay. Saliva was collected in the Oragene OG-500 collection kits. DNA was extracted manually using the prepIT-L2P DNA Genotek (catalog # PT-L2P).

### Pathology

Women from Nigeria with BrC were clinically staged according to the American Joint Committee on Cancer system [33]. Immunohistochemistry of the primary tumors was done with monoclonal antibodies for estrogen receptor (ER), progesterone receptor (PR) and HER/2neu (HER2) and Ki-67, EGFR and CK5/6 using monoclonal antibodies from Thermo Scientific™(clones ER-SP1; PR-SP2; HER2-SP3) and Thermo Scientific™ Ultravision™ Quanto Detection System HRP DAB detection kit according to the manufacturers recommendations.

### Polymerase chain reaction

- **PCR to search for Xq21.33 was carried out by screening with the following primers:**
- **Coff Xq21.33** ACG CTG TTT TGT CCC TTT GA.
- **1016 R** CGTCGACTTGTCCTCAATGACCACGCT.
- 1**584R** TTTGCGTTGACT*GAG*CCATTACCG.
- **2477 R** TTG AGC AAC ATC TTG *GAG* CCT TGC.
- **3456R** ACT TGC CCA ATA TGC AGC CTT TCC TCC.
- **6115R** TCA ACT ATG GCC TGT CCT TGC GAA.
- **5355F** TCG GCT CAA AGA GCA *GAG* ATG GTT.
- **F Flank** TCAGTC TGA AGA CAA TGA CAT ACT T T.

Approximately 200 ng of DNA was tested with a primer Coff Xq21.33 in the 5′ insertion site and any second primer (either 1016R, 1584R, 2477R, 3456R, or 6115R) located in the Xq21.33 genome, and/or a 3′ insertion site primer, F Flank (R) to 5355F in the virus. PCR was carried out using the LongAmp® *Taq* PCR Kit (New England Bio Labs). Reactions were carried using 5ul of 5X Long Amp Taq reaction buffer + .75ul of 10uM DNTP, + 0.2 ul of 50uM forward and 0.2ul of 50uM reverse primer with addition of 1ul of Long Amp Taq DNA polymerase plus the addition of between 50 to 250 ng of sample DNA to a final volume of 25ul. In general, the reactions were carried out 94 °C initial denaturation for 30 s followed by 35 to 40 cycles of 94° for 15 to 30 s, 55° to 60° for 15 to 30 s and 65° for 2 to 5 min, and then final extension at 65° for 10 min. All positive samples to 1011R to Coff Xq21.33 were confirmed with at least 3 other primer sets.

Homozygosity was determined by using primers at Xq21.33 and F Flank to obtain a PCR product of 420 bp in heterozygotes and no amplification product in homozygotes.

### Genotyping of Xq21.33

Genotyping of the 3′ *env* region of Xq21.33 was carried out by creating an amplified product using F Flank to 5355F using the protocol noted above. The amplified product was resolved from 0.8% agarose gels and bands of DNA of interest were cut from gels and purified using the QIAquick Gel extraction kit QIAGEN according to the method of the manufacturer. The purified product was sent for direct sequencing to the DNA sequencing core of the University of Michigan using primers in forward or reverse orientation ranging from 5350 to 9472.

### Phylogenetic analysis

Neighbor-Joining trees were generated using MEGA 7. The evolutionary history was inferred using the Neighbor-Joining method [34]. The percentage of replicate trees in which the associated taxa clustered together in the bootstrap test (1000 replicates) are shown next to the branches [35]. The tree is drawn to scale, with branch lengths in the same units as those of the evolutionary distances used to infer the phylogenetic tree. The evolutionary distances were computed using the Maximum Composite Likelihood method [36] and are in the units of the number of base substitutions per site. The rate variation among sites was modeled with a gamma distribution (shape parameter = 1). There were a total of 3044 positions in the final dataset. Evolutionary analyses were conducted in MEGA X [37].

### Statistical analysis

Differences in the presence of the Xq21.33 provirus between cases and controls were examined using Fisher exact tests All analyses were performed using Stata SE version 15.1 (College Station, Texas). A type I error (α) level of 0.05 was considered significant for testing the study’s hypotheses. http://www.stata.com/support/faqs/statistics/adjusted-means-after-anova.

## Results

Two hundred thirty-nine Nigerian women with BrC participated in this study. In 4 the DNA concentration was too low for adequate use in PCR. In addition, in 19 consecutive samples there was an interfering substance present which prevented PCR reactivity. These samples were amplified multiple times with different primers to get a signal, but they would not amplify. These patients are not included in this study. This left 216 women with breast cancer who became part of this study. There were 229 control women enrolled in the study. Of these, 10 had very poor DNA content. This left 219 control women as part of this study.

**Fig. 1:**
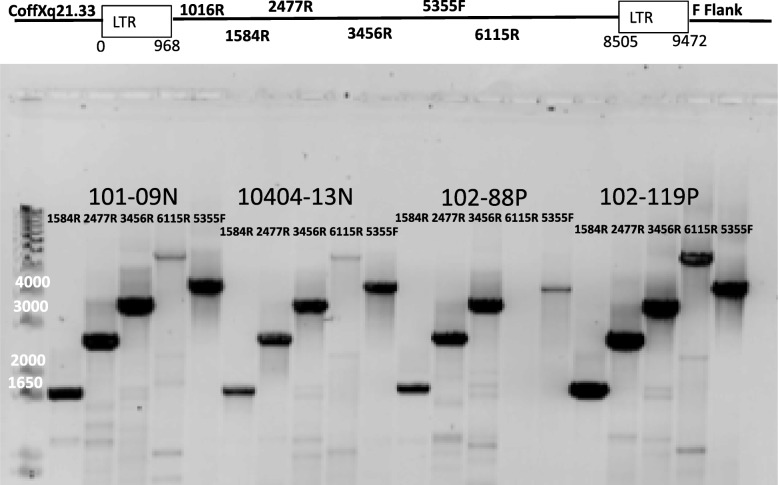
This shows the PCR product from amplifying the DNA of the Xq21.33 proviruses from two Nigerian women without breast cancer (labeled N) and two women with breast cancer (labeled P). The primers sets were CoffXq21.33 to 3 reverse primers noted and one set from 5355F to F Flank which spans the *env* region of the provirus

Proviral DNA was screened for in all samples first using the primer set 1016R and Coff Xq21.33. All positive samples were then screened with other primer sets including 1584R, 2477R, 3456R and 6115R in conjunction with Coff Xq21.33. This covered the LTR and the *gag* and *pol* genes of the provirus. In addition, we screened positive samples for the *env* gene using 5359F and F Flank. Representative positive samples are shown in Fig. 1.

The Xq21.33 provirus was present in 27 of 216 (12.5%) Nigerian BrC cases compared to 22 of 219 (10.0%) controls These results were not statistically different.

Table 1 presents the study participants by age groups and by virus status. We did not have accurate ages on all women. In those with ages specified, as seen in Table 2, in women with BrC who were 44 years and younger (presumed to be premenopausal) the virus was found in 12 of 117 (10.3%), while it was in 18 of 157 (11.5%) control women under age 44 years. Interestingly, this difference breaks apart in women older than 44 years (presumed to be postmenopausal), 15/98 (15.3%) women with BrC were virus positive while in women without BrC only 4/54 (7.4%) were virus positive. This difference was not statistically significant at the .05 level; the p- value was (0.079), but is suggestive of a trend that may warrant further exploration if the sample size of older women was higher.

**Table 1:** Prevalence of Xq21.33 Detection in African Women with or without Breast Cancer

Age	BrC No Xq21.33	BrC Xq21.33	No BrC No Xq21.33	No BrC Xq21.33	(chi square test, *p* value)
15–19	0	0	1	0	
20–24	2	0	3	1	
25–29	12	0	25	3	
30–34	17	4	31	5	
35–39	38	4	38	4	
40–44	36	4	41	5	
Total pre-menopausal	105	12	139	18	X^2^ = 0.100 *p* < 0.3757 (ns)
45–49	23	3	26	3	
50–54	27	8	8	0	
55–59	16	2	11	1	
60–64	7	1	5	0	
65–69	6	0	0	0	
70–74	1	0	0	0	
75–79	3	0	0	0	
80–84	0	0	0	0	
85–89	0	1	0	0	
Total post-menopausal	83	15	50	4	X^2^ = 1.986 *p* < 0.079 (ns)
Age unknown	1		8		
Total	189	27	197	22	

Detection of HK2 Xq21.33 provirus in the DNA of BrC and control women (No BrC) in populations from African ancestry. The *P* values show the statistical significance difference of Xq21.33 virus detection between pre-menopausal and post-menopausal women with and without BrC. The statistical test Chi-square without Yates correction was used. The X^2^ and p values are indicated. There is no statistical difference between the prevalence of Xq21.33 in the pre-menopausal women but the prevalence of Xq21.33 in post-menopausal women is approximately double that in women without breast cancer but this is also not statistically different at a *p* value of <.05

**Table 2:** Prevalence of Xq21.33 in different populations

	Caucasian	African	African American	(chi square test, p value)
BrC	0/95			
No BrC	0/8		0/9	
Ghana BRC		6/87		
Nigeria BrC + no BRC		49/435		
Pygmy		1/6		
African American BrC (women)			6/51	
African American ^a^PCa (men)			3/42	
HIV+	0/6		1/3(men) 0/8 women	
Total	0/109 (0%)	56/528 (10.6)	10/113 (8.8%%)	

^a^*PCA* Prostate Cancer

Detection of HERV-K HML-2 Xq21.33 virus in the DNA of populations from African and Caucasian ancestry. The P values shows the statistical significance difference of Xq21.33 virus detection between Caucasian individuals and population from African ancestry. The statistical test Chi-square without Yates correction was used. The X^2^ and p values are indicated. Caucasian Vs African, (X2 = 16.094, *p* < 0.0001). Caucasian Vs African American, (X2 = 14.959, p < 0.0001)

We used this age 45 or greater as presumed postmenopausal age as we have broken our data into age groups at 5 years intervals. In Nigeria the average age of menopause is 48.4 +/− 5 years (samples size *n* = 563 age range 44–87) [38], and in Ghana the average age of menopause is 48.4+/− 3.62 (sample size *n* = 123 age range 40–56) [39].

We next looked at DNA from patients in the United States who had BrC and other conditions (Table 2). Of note, 10/113 (8.8%) African Americans were virus positive. Of these 6/51 (11.8%) were women with BrC, 3/42 men with prostate cancer, 1/3 men and 0/8 women with HIV having virus and 0/9 persons with other cancers. No Caucasian American patients had virus, (*n* = 109) confirming previous observations reporting the specific incidence of Xq21.33 in in people with African ancestry [31]. We also screened buccal swabs collected from patients from Ghana. 6/87 (6.9%) had provirus in contrast to 49/439 (11.2%)in Nigeria and 1/6 (16.6%) among Pygmies from the Congo basin.

**Fig. 2:**
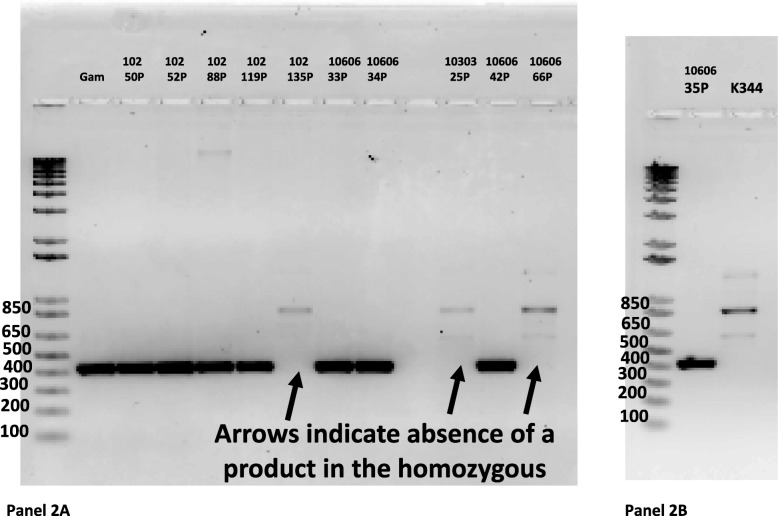
Panel **a** shows a representative agarose gel to detect homozygocity or hemizygocity in African female individuals with BrC and HERV-K HML-2 Xq21.33 virus. Amplicons from DNA from some women with BrC (#P) and a normal Gambian person are shown which were obtained using a primer in the preintegration region of Xq21.33 on the 5′ side of the virus (CoffXq21.33) and a primer on the 3′ side of the integration site (F Flank). Homozygotes would not give a product as the 9472 bp virus present in both chromosomes X will not allow amplification using this protocol whereas hemizogous (having HERV-K HML-2 Xq21.33 in only one chromosome X) would give a 480 bp product corresponding to the empty preintegration site of one chromosome X. Panel **b** shows a male K344 who has HIV lymphoma and has the Xq21.33 virus present. This virus is only in his X chromosome while his y chromosome has no virus. His DNA would not give a 480 bp amplicon using this same protocol. A patient with breast cancer who is hemizygous is shown in the 1st lane next to the molecular weight markers

We looked to see if there was homozygosity/hemizogocity for this virus in women with BrC and virus vs those without breast cancer and provirus. An illustrative PCR gel is shown in Fig. 2. We found that 5/27 (18%) women with breast cancer were homozygous for provirus while 2/22 (9%) women without breast cancer and provirus were homozygous. This was not statistically significant.

We were particularly interested in seeing if the pathology of BrC was different in women who had the virus and those who were negative. Extensive biomarkers were carried out on the Nigerian cohort of women as shown in Table 3. As can be seen there was no distinguishing difference in the biomarker status between the patients who were virus negative vs those who were virus positive. Triple negative BrC was rather common in this population occurring in 49/156 cases (31.4%). The ages of women who were triple negative were not different from those who had other hormonal markers tested.

**Table 3:** Hormone Receptor Status in Breast cancer specimens

	Virus Negative	Average Age	Virus Positive	Average Age
Not done	51	43.2	5	43.8
ER-, Pr-, Her2Neu-	43	46.4	6	43.8
ER+, PR+ Her2Neu-	25	45.3	4	59
ER+, PR+, ER2Neu+	11	40.9	1	38
ER+, Pr-, HER2Neu-	17	45.1	2	40.5
ER+, Pr-, HER2NEU+	3	43.6	0	N/A
ER-, PR- HER2Neu+	12	45.9	4	49
ER-, PR+, HER2Neu-	6	48.2	1	52
ER-, PR-,HER2Neu ND	4	34.5	2	34
ER-. PR+, HER2Neu+	1	28	1	58
ER+, PR ND, HER2NEU ND	6	45.2	0	N/A
Other	6	44.5	1	52

In the virus positive group 3/27 are read as Ductal Carcinoma in situ

**Fig. 3:**
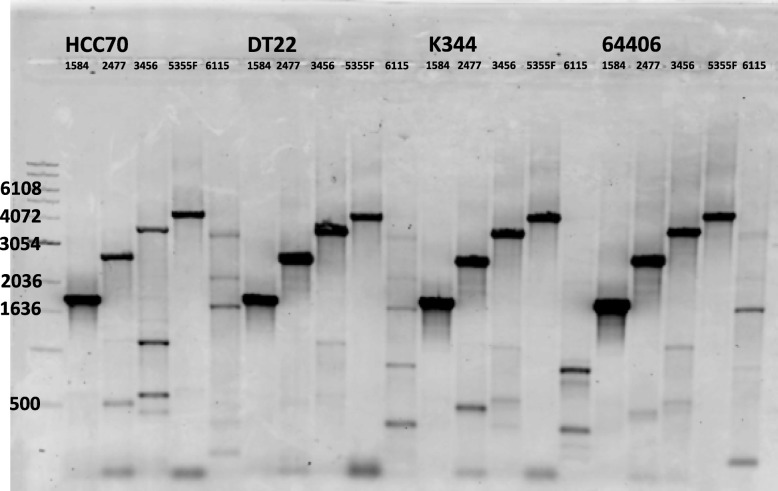
Detection of HERV-K HML-2 Xq21.33 in cell lines and African male descendants. This shows the products of amplification of DNA from two Breast cancer cell lines HCC70 and DT22 and K344 from an African American man with HIV associated lymphoma and an African American man with prostate cancer. The primer sets used march the genome of Xq21.33 using Coff Xq21.33 F to 1584 R, 2477R, 3456R and 6115R and another primer set 5355F to F Flank. The laddered bands represent the appropriate amplicon for these primers. Note that the 6115R to Coff Xq21.33 did not amplify as it is often difficult to produce a product because of the large size of the amplicon. A nonspecific amplicon is seen in the 6115 lane

We also screened known cell lines for the presence of Xq21.33 proviruses as shown in Table 4. Two of these 28 cell lines, 2 (DT 22 and HCC70) were established from triple negative BrC arising in African women. Representative screening for the virus is shown in Fig. 3.

**Table 4:** Detection of Xq21.33 in Cell lines

Cell Line	Xq21.33	Ethnicity	Cell Type	Gender
HCC70	+	Black	Primary duct cancer	female
DT22	+	Black	Triple negative	female
MCF7	–	Caucasian	Adenocarcinoma breast	female
T47D	–	unknown	Metastatic pleural effusion	female
SKBr3	–	Caucasian	Invasive duct cancer	female
K151	–	Caucasian	Invasive duct cancer	female
BT474	–	Caucasian	Invasive duct cancer	female
HUS78	–	unknown		unknown
MDA MB453	–	Caucasian	Adenocarcinoma breast metastatic pleural effusion	female
MDAMB 157	–	Black	Medullary carcinoma breast	female
MDA MB468	–	Black	Adenocarcinoma pleural space (breast)	female
HCC 1806	–	Black	acantholytic squamous cell carcinoma breast	female
SUM149	–	NA	Triple negative Invasive duct BrC	Female
ZR7530	–	Black	Invasive duct BrC	female
JURKAT tAT	–	unknown	acute T lymphoblastic leukemia	male
DHL1	–	unknown	Diffuse large cell lymphoma cell line from pleural effusion	male
HL60	–	Caucasian	Acute promyelocytic leukemia	female
HUT 78		Caucasian	Cutaneous T cell lymphoma	male
H9	–	Caucasian	Derivative of HUT 78	male
SupT1	–	Caucasian	T lymphoblastic lymphoma	unknown
MT4	–	unknown	Adult T acute lymphoblastic leukemia	male
09–1521	–	unknown	EBV+ B cell from follicular lymphoma	unknown
12–1697	–	unknown	EBV + B cell from lymphoma	unknown
12–135	–	unknown	EBV negative cell line from marginal zone lymphoma	unknown
13–1357	–	unknown	EBV negative cell line from Hodgkin lymphoma	unknown
14–130	–	unknown	EBV+ cells from follicular lymphoma	unknown
15–107	–	unknown	EBV - cells from transplant lymphoma	unknown
NSUH1	–	Caucasian	EBV – cells from diffuse large B cell lymphoma	male

We did genomic sequencing in order to determine if there is variation in the Xq21.33 sequence in populations from West Africa. Mutations found from PCR product amplified from primers 5355F to F Flank giving a sequence spanning the *env* region of the virus are shown in Table 5. Two sets of complete sequences spanned long regions. In one set we sequenced from approximately 5390 to 9472 and in a second set we extended this from approximately 5355 to 9472. In a third set we could not get complete sequences and had areas of approximately 50–150 bp long which lacked adequate sequences. Using 30 complete sequences from 5355 to 9472, all had a common mutation of 5370 T to A, and both sets of complete sequences (total 46) had a mutation of 8529 A to G. Twenty five of 46 samples (54.3%) had a mutation of 7596 T to C when compared to the deposited sequence KU054272.1. Samples from Nigerians, Ghanaians and African Americans had these same 3 mutations. The two cell lines DT22 had HCC70 both had the 5370 and 8529 mutation present, while DT22 also had a 7596 mutation. The 5370 or 8529 may be true SNPs, but also may just represent an error in the original sequence. There were 2 Nigerian and 2 Ghanaian sequences found with both having the 5370, 8529 and or the 7596, while other mutations were found as shown in Table 5.

**Table 5:** Mutations in the *env* region of HERV-K HML-2 Xq21.33 (Acc. No. KU054272.1)

Possible first conversion events	
5370 T to A Nigerian, Ghanaian, African Americans, Pygmy Cell line HCC (found in complete sequences from 5355 to 9472)	30/30
8529 A to G Nigerian, Ghanaian, African Americans, Pygmy Cell line HCC and DT22 (found in complete sequences from 5355 to 9472 and from about 5390 to 9472)	46/46
7596 T to C Nigerian, Ghanaian, African Americans, Pygmy Cell line DT22 only	25/46
Possible second conversion events	
Nigerians only 5370, 8529 and 7596 + 5735 C to T, 6089 C to T, 6345 C to T 7274 A to G, 7795 G to A, 8914 T to C	2/2
Ghanaians only 5370, 8529 and 7596 + 6914 C to T, 7203 G to A, 8170 A to G, 8600 C to A	2/2

Genetic sequences were compared to the known HERV-K HML2 sequences to understand the evolution of Xq21.33 and how 2 Nigerians and 2 Ghanaians had more than 3 SNPs present. All proviruses had the 5370 and 8529 mutation, which may have existed in the founder virus, while about 54.3% of proviruses had an additional mutation at 7596. All mutations preserved the integrity of the *env* protein.

**Fig. 4:**
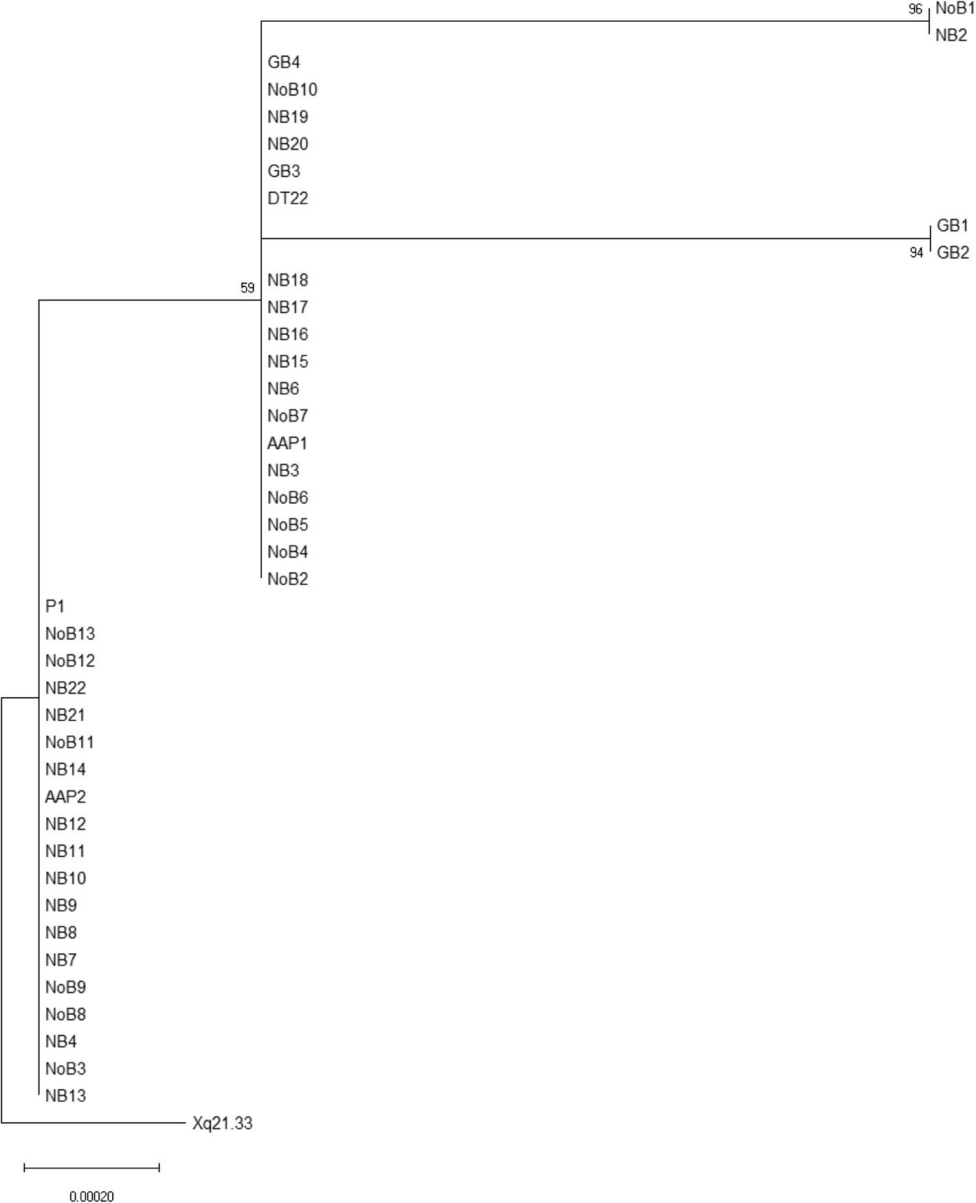
Evolution of HERV-K HML-2 Xq21.33 in Africa. Evolutionary relationships of taxa**.** The evolutionary history was inferred using the Neighbor-Joining method [34]. The optimal tree with the sum of branch length = 0.00263695 is shown. The percentage of replicate trees in which the associated taxa clustered together in the bootstrap test (1000 replicates) are shown next to the branches [35]. The tree is drawn to scale, with branch lengths in the same units as those of the evolutionary distances used to infer the phylogenetic tree. The evolutionary distances were computed using the Maximum Composite Likelihood method [37] and are in the units of the number of base substitutions per site. The rate variation among sites was modeled with a gamma distribution (shape parameter = 1). This analysis involved 42 nucleotide sequences. Codon positions included were 1st + 2nd + 3rd + Noncoding. All ambiguous positions were removed for each sequence pair (pairwise deletion option). There were a total of 3044 positions in the final dataset. Evolutionary analyses were conducted in MEGA X [36]. The tree is rooted to the known Xq21.33 HERV-K HML-2 sequence (Accession no. KU054272.1). NB: Nigerian BrC, NoB: Nigerian No BrC, AAP: African American PCa, P: Pygmy, GB: Ghanaian BrC, DT22: African BrC cell line. The left side of the tree would indicate a first genetic drift (T > C) event and at the right side the three show two small protruded branches that indicate gene conversion events

We performed a phylogenetic analysis to understand the evolution of HERV-K HML-2 sequences isolated in Nigerian and Ghanaian populations. A Neighbor-Joining tree shows that genetic mutations and possible gene conversion events occurred during HERV-K HML-2 Xq21.33 evolution (Fig. 4). Placing Xq21.33 reference sequence as the ancestral sequence, the tree revealed two different features. First, a genetic mutation (T > C) of Xq21.33 appeared in sequences of Nigerian and Pigmy populations in West-Central Africa, but also in all sequences obtained from Ghana (West Africa) (Supplementary Fig. 1). This suggests that the spread of this mutation may have occurred from the migration of individuals in this area of Africa. The tree also shows two Nigerian and two Ghanaian individuals (NoB1, NB2, GB1 and GB2 respectively) who have additional mutations, (Supplementary Fig. 1) likely originated by gene conversion between Xq21.33 and other HERV-K HML-2s. These HERV-K HML-2 mutations were found in HERV-K HML-2s in other chromosomal locations. The divergence between HERV-K HML-2 Xq21.33 sequences in African populations appears to indicate that these events happened relatively recently in human evolution. Further evidence of gene conversion may be a result of copying HERV-K HML-2 Xq21.33 from one X chromosome to the other. Although it is possible that the appearance of homozygous inherited HERV-K HML-2 Xq21.33 may have come from both parents, this probability is very small, considering the rare frequency of Xq21.33, which in these screened populations in Africa was 10–12% in women and probably much lower in men. This would make the predicted homozygous state to be less than 1%, whereas in these females the prevalence of being homozygous was 24%, which is more likely the ¼ chance that can occur during meiotic recombination. Therefore, it is more likely one HERV-K HML-2 Xq21.33 virus copied to the other chromosome X during meiosis by gene conversion.

## Discussion

To date studies of endogenous retroviruses have been trying to determine if any of these proviruses can be activated again to produce infectious virions which may genetically alter cells and lead to a disease process. Thus far no provirus has been convincingly associated with a disease. Great interest has been placed on seeing if any or some HERV-K HML-2 viruses can be linked to BrC, as these viruses are genetically distantly related to MMTV. Early studies of BrC cell lines revealed the presence of viral particles possibly related to HERV-K HML-2. The T47D cell line grown from a neoplastic pleural effusion was shown to produce viral particles when stimulated first with estradiol and then progesterone [40]. At first these particles were thought to be more related to MMTV [40–42]. Further studies of the T47D cell lines [43, 44] and cells grown from BrC pleural effusions [45] further suggested that a similar MMTV-like virus called HMTV was seen in such cell cultures [45]. Later it was felt these were more likely related to HERV-K HML-2 proviruses [46].

Attempts were made to determine if HERV-K HML-2 proteins might appear in BrC cell lines or tumors. Indeed NP9, a 9 kb protein expressed predominantly in the nucleus as a product of Type 1 HERV-K HML-2 proviruses, was described by Armbruester et al. [47]. This protein was found in 4 BrC cell lines, (T47D, MCF7, MDA-MB231 and SKBr), as well as in 52% of BrC tumors. Transcripts of HERV-K HML-2, mostly from type 1 proviruses, could also be detected in most BrC cell lines and tumors [48]. Type 2 transcripts which might express the envelope of HERV-K HML-2 were also found, [49] and in different cell lines hormonal stimulation increased the mRNA expression of HERV-K HML-2 transcripts. Similarly, increased expression of HERV-K HML-2 was reported as high as 70% of tumors. Both type 1 and type 2 transcripts were seen. Monoclonal antibodies were prepared to the recombinant fusion protein K10Q18, which resulted in a monoclonal antibody with great affinity for the K SU, which is a 55-kDa surface subunit of HERV-K HML-2 rather than the 39-kDa transmembrane subunit *env* surface protein [50]. Eighty-five percent (85%) of invasive ductal carcinoma (IDC) biopsies showed expression of this protein, while only 28.57% of normal breast tissue and/or hyperplastic breast tissue showed such protein expression. Most of the protein was expressed in the cancer tissues [51]. Antibodies to the SU protein, using immunoprecipitation and enzyme linked immunosorbent assay (ELISA) technology, were seen in 50% of samples, some with rather high titers. Only low titers of antibody were seen in normal controls, suggesting that antibody levels might be associated with cancer progression. However, a different study of antibodies to the SU fragment failed to show any distinguishing antibody response between normal tissue and BrC cells [52]. Various immunoassays, including Interferon γ (IFN-γ) ELISPOT assays, revealed “that PBMCs from BrC patients contain HERV-K specific cytotoxic lymphocytes (CTL) that can be re-stimulated in vitro to induce detectable cytolytic activity against HERV-K expressing targets” [51]. Similarly, these same PBMCs from BrC patients showed HERV-K HML-2 specific cytokine secretion against HKL2 target cells. In addition, “anti HERV-K specific monoclonal antibodies inhibited growth and induced apoptosis of BrC cells in vitro” in a mouse xenograft model using different cell lines [53]. Human tumors showing more expression of HERV-K HML-2 SU antigen on their surface were more likely to have metastatic potential. Further studies showed again that there was high expression of e*nv* protein on tumors of both American and Chinese patients, and the level of expression correlated with poor prognosis [54]. Using The Cancer Genome Atlas (TCGA) RNA Seq-database to evaluate the “expression of the HERV-K 108 (7p22.1), HERV-K 109 (6q14.1) HERV-K 113 (19p12b) and HERV-K 115 (8q23.1) loci in basal, HER2E, LumA and LumB breast cancer subtypes”, it was found that in 512 patients, HERV-K HML-2 from these 4 different loci were more highly overexpressed in this type of BrC [55]. Subsequent studies showed that activation of HERV-K *env* was necessary for metastasis and tumorigenesis of BrC cells [56].

Other studies have looked to see if H2K proviruses are involved in transcription during transformation of mammary epithelial cell to neoplastic cells. This was accomplished by taking human mammary epithelial cells and immortalizing them through telomere maintenance by “hTert” (encoding human telomerase reverse transcription) over expression. These cells were then transformed with SV40 small and large T antigen, and then further transformed with a murine leukemia virus (MLV) base vector. This led to over expression of the BrC oncogene HRAS (V12) and/or ERB2 (Her2/neu). These cells were then looked at to see what mRNAs were used that belong to the HERV-K HML-2 group. Surprisingly, the HML2 transcriptome analyzed showed that most of transcription arose from 15 HML2 proviruses. The majority of transcription was in an antisense orientation and “from mostly older proviruses integrated within introns” [57]. Viruses that transcribed mRNA in a sense orientation did not produce transcripts that could make any proteins, which is contrary to what has been described in BrC cell lines and even in tumors themselves.

With all of this analysis so far, it still remains unclear if there is a specific HERV-K HML-2 provirus associated with BrC and/or how activation of HERV-K HML-2 might be playing a role in formation of BrC. With the discovery of the new Xq21.33 provirus [31], which appears to be present in limited populations only in Africa, and mostly in Nigeria and Gambia and in Pygmy populations, it seemed that a study of BrC arising in patients carrying Xq21.33 could provide insight into whether having this provirus might predispose a person to BrC, especially as the Xq21.33 virus is fully able to make all viral proteins. We thus embarked on this study of women with BrC in Nigeria and compared the prevalence of this provirus to a control group of women without BrC. We also studied the distribution of this virus in other areas of Africa and the United States.

In Nigerian women the prevalence of this virus is higher (49/444 women 11.03%) than that detected by Wildschutte et al. (6.9%) [31]. This difference may occur because we had more persons to test, and their data was from the 1KGP which has a very different sampling method. We show that this provirus is present not only in Nigerian populations, but also in Ghanaian women, Pygmy people from Gambia, and not surprisingly, also in African American men and women (10/113 8.8%) whose heritage dates back to these countries.

This provirus has not spread as widely as younger proviruses K113 or K115, which have been reported to be present in 21.8 and 34.1% of African populations [28]. For instance, sequence analysis shows that Xq21.33 is likely younger than K113 and K115, and a single mutation (T > C, base 7596) can predict to some degree human migration from Central to West Africa 100,000 to 200,000 years ago. Then, it appears that Xq21.33 is the youngest HERV-K HML-2 virus in humans. In addition, the Xq21.33 viral sequence incorporates few additional mutations by gene conversion, likely due to its localization in Chromosome X, where it can exchange material with the complementary X chromosomes during meiosis. Finding signatures of Xq21.33 from HERV-K HML-2 residing in other chromosomes also suggest that Chromosome X HERV-K HML-2 may exchange material with other somatic chromosomes, although at a low rate. Considering the young age and active sequence area in Chromosome X, it is possible that Xq21.33 produces some level of increased morbidity and mortality in those who have virus, which hinders the spread of virus over the generations.

These SNPs found in HERV-K HML-2 Xq21.33 do not create stop codons in the viral *env* region but might modify the biology of the provirus. Several samples have multiple SNPs which, on genetic analysis, suggests some form of gene conversion occurring in some Nigerians and some Ghanaians. The mutations we found cannot be explained by PCR artifact which would produce many random mutations, rather than those seen. This would suggest that Xq21.33 is in a hot spot of the genome, which may allow for some recombination with other active viruses, such as K113.

There was no difference in the prevalence of virus between those women with BrC and women without BrC indicating that this virus does not play an active role in breast carcinogenesis. In an unplanned analysis by menopausal state if one divides these patients into pre and post-menopausal groups, then virus is more prevalent in post-menopausal women, rising from 12/117 or 10.25% to 15/98 or 15.30% in women with BrC while falling from 18/157 or 11.4% to 4/54 or 7.4% in post-menopausal women without BrC. This difference in frequency of provirus in post-menopausal women is not statistically significant at the 0.05 level as the study was not powered to detect such small differences, should they be relevant to the process or carcinogenesis, but is closer to the 0.079 level. It is clear that our sample size is too small. Clearly an adequately powered study of post-menopausal women might be undertaken to explore a potential significant association of this virus in post-menopausal African and or African American women with BrC.

We did see a higher prevalence of triple negative BrC, as has been reported earlier in patients from Africa. Our patients are relatively younger than patients with BrC in Europe and the Americas. This may be more due to the fact that the average life span of women living in Nigeria, according to the World Health Organization, is about 54.7 compared to that in the United States, Germany, Sweden and the United Kingdom, which is about 81.0, years of age. As health is improving in Nigeria the percentage of triple negative BrC may reduce, as older women are less likely to have triple negative cancers.

In order to understand the potential role of this virus even if our study, or a future larger study with more post-menopausal women, had shown a statistically relevant association of Xq21.33 in post-menopausal BrC, it would still be necessary to show that this provirus has activated and is now in some area in the BrC cell different from Xq21.33 and the known HERV-K HML-2 integration sites and in a genomic area where it might disrupt some cellular growth factor. Such a virus would also have to be able to infect breast tissue. Recently it has been shown that the HERV-K HML-2 *env* gene from the artificially constructed Phoenix virus, [58] encoded in a vesicular stomatitis virus, will bind to heparan sulfate molecules which are universally present on many cells [59]. It was also shown that an Xq21.33 *env* construct similarly binds to heparan sulfate [59]. Heparin sulfate binding sites are common on many cells. If there is an infectious HERV-K HML-2, then it could readily infect a person exogenously through skin and/or via blood exposure, or it could activate from the genome.

Since cells have 117 proviruses present, as well as over 2000 LTRs and over 1000 pericentromeric and centromeric HERV-K HML-2 s, all of which are almost 97–99% the same, using current platforms for sequencing to detect provirus would be very difficult as a newly integrated provirus would be obscured by such background noise. One would have to find sequences that are within a new area of the genome and or repetitive elements in the centromeric regions. Such a sequence would have to span the full LTR and extend into the sequence of the virus itself. Current sequencing provides only fragments of 300–400 bp, which are not of sufficient length, or number, to distinguish virus from background HERV-K genomes and LTRs. However, if virus did get to the breast and integrate in an area to induce an oncogenic change, virus would be amplified many fold by the clonal expansion induced by the cancer. This new insertion site in the cancerous breast tissue could only be detected by sequencing larger portions of the genome with new available technologies that give long sequence reads. Similar technology has been used in a study of large granular cell leukemia thought to be due to a HERV-K HML-2 virus. While this study could not confirm the presence of a HERV-K-HML-2 virus it does provide insight into how such technology can be used in cancerous cells [60].

## Conclusion

We searched for evidence that a recently discovered HERV K HML-2 virus Xq21.33 might play a role in predisposing women to BrC in those carrying this provirus. Our data describes some of the genetic variation in this provirus but our data could not support an association of Xq21.33 with breast cancer. However, it does suggest that there may be some role for this provirus in predisposing post-menopausal women carrying this virus to breast cancer which could be discovered if a more appropriate sample size of older patients was studied. Finding of such an association would be important as antiretroviral drugs might be useful in preventing BrC in such women.

## Supplementary information

**Additional file 1: Supplementary Fig. 1.** Alignment of HK2 X21q33 sequences from populations of African ancestry.

## Data Availability

All data is available from the principle author Mark H. Kaplan MD including PCR gels and sequencing data on reasonable request from mhkaplan@umich.edu .

## Abbreviations

1KGP: One thousand genomes project
BrC: Human breast cancer
ELISA: Enzyme linked immunosorbent assay
ER: Estrogen receptor
HER 2: HER/2neu
HMTV: Human mammary tumor virus
IRB: Institutional Review Board
LTR: Long terminal repeat
MLV: Murine leukemia virus
MMTV: Mouse mammary tumor virus
MYA: Million years ago
PBL: Peripheral blood mononuclear cells
PCR: Polymerase chain reaction
PR: Progesterone receptor
SNP: Single nucleotide polymorphism
TCGA: The Cancer Genome Atlas
WGS: Whole genome sequencing
