# Supplementary figures and images for: Is the HERV-K HML-2 Xq21.33, an endogenous retrovirus mutated by gene conversion of chromosome X in a subset of African populations, associated with human breast cancer?
Source: Infect Agent Cancer. 2020 Mar 7;15:19. doi: 10.1186/s13027-020-00284-w (PMC7060579; doi:10.1186/s13027-020-00284-w)

Supplementary figure 1. Alignment of HK2 X21q33 sequences from populations of African ancestry
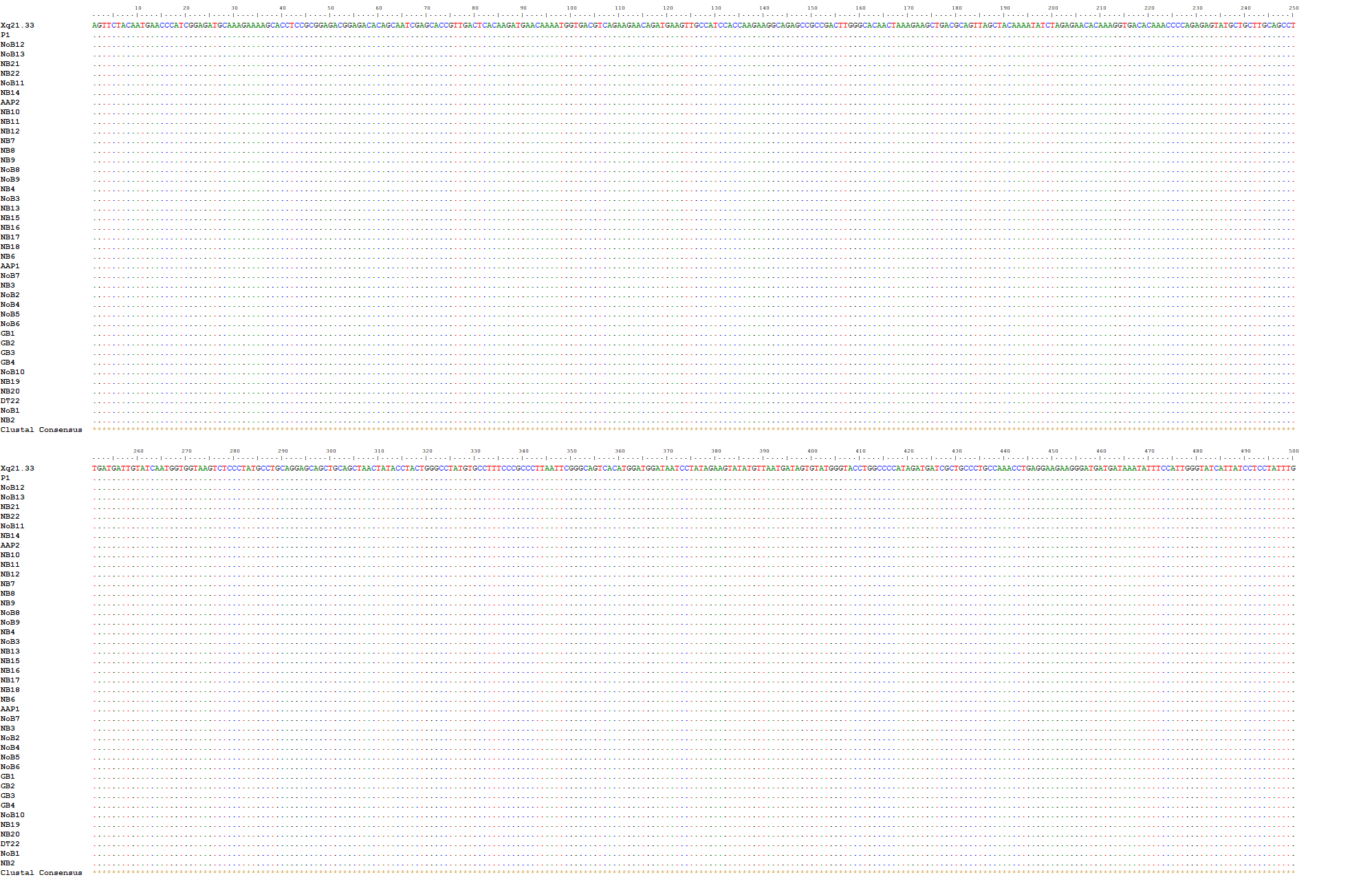


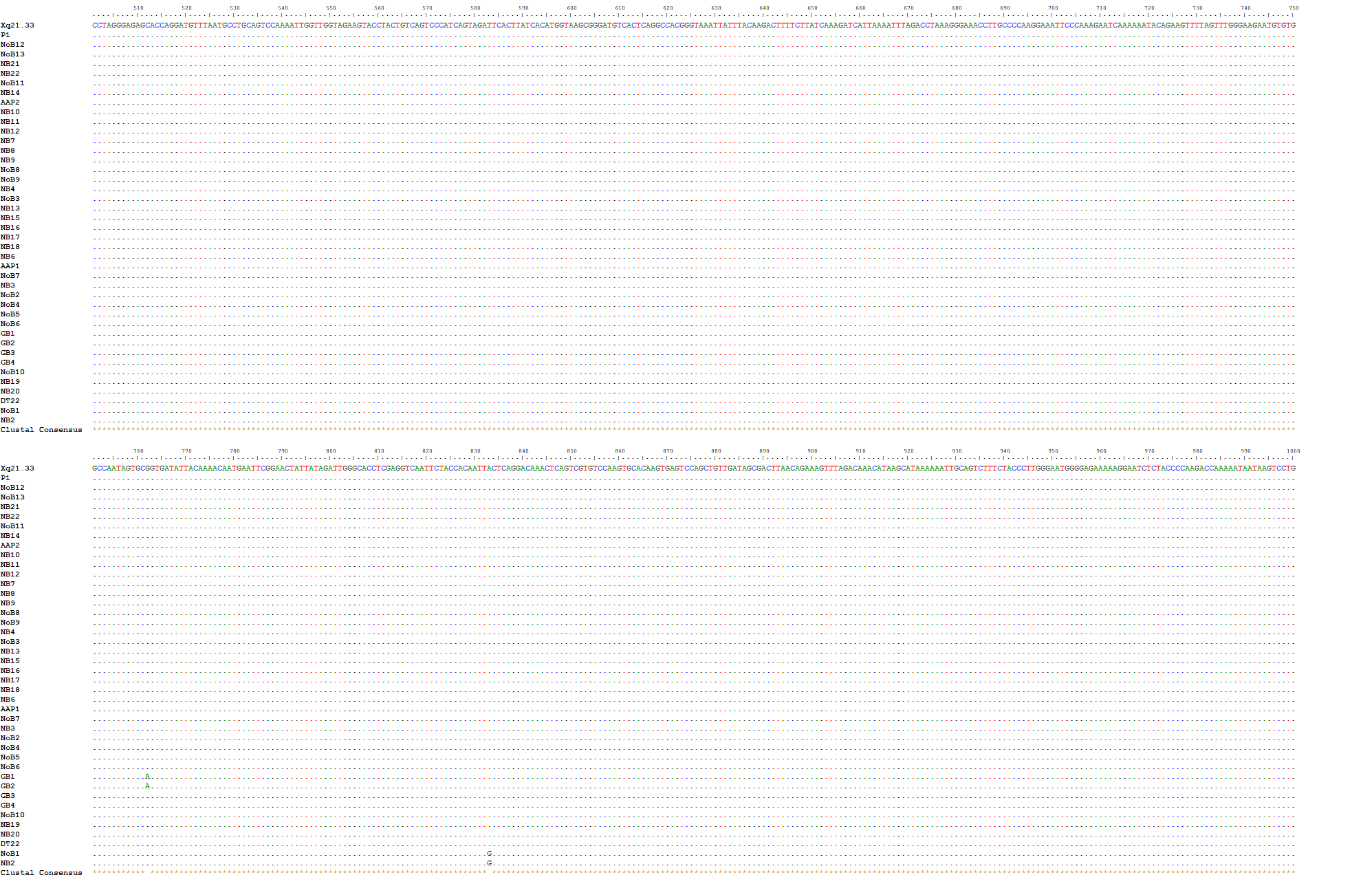


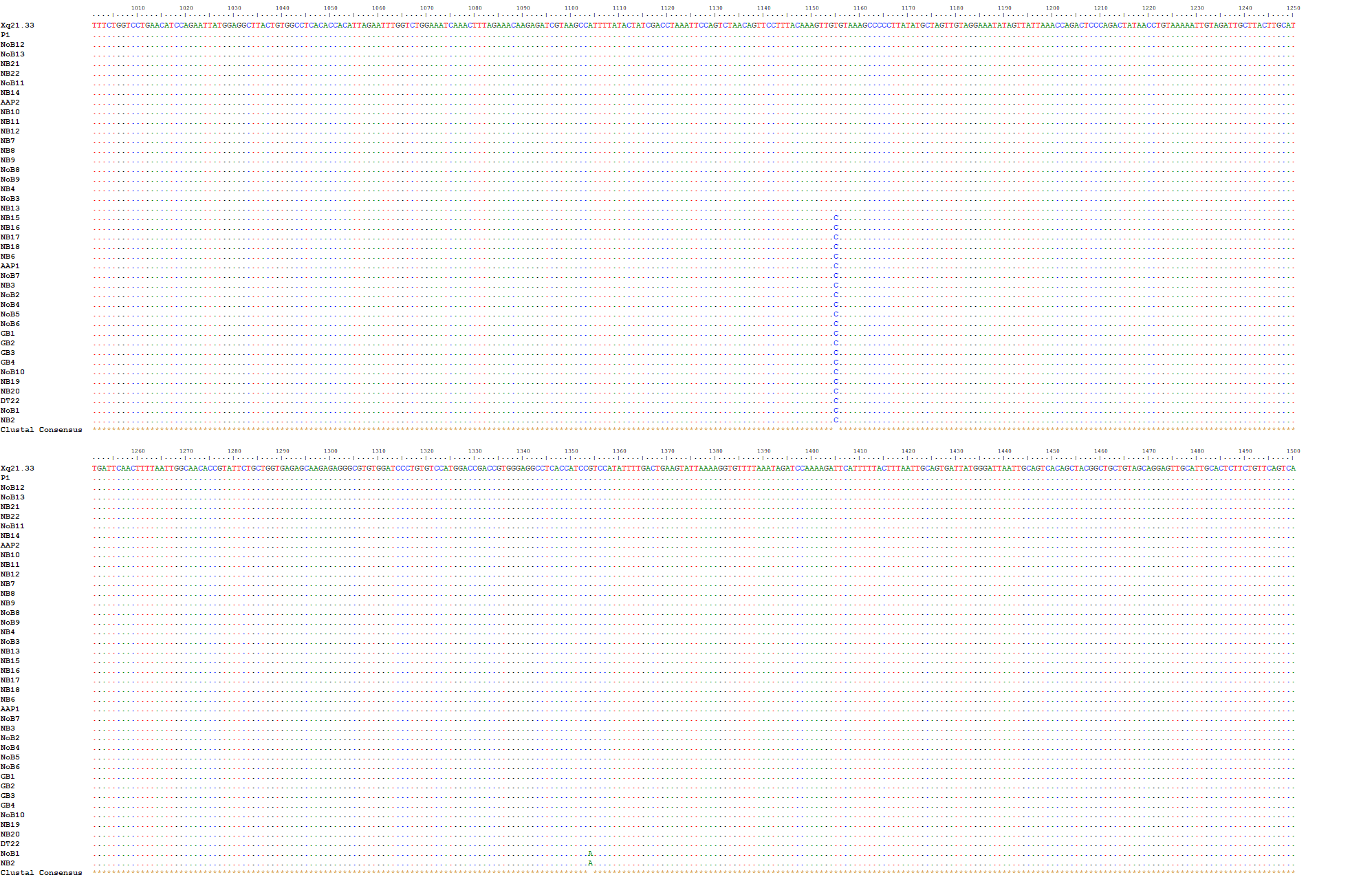


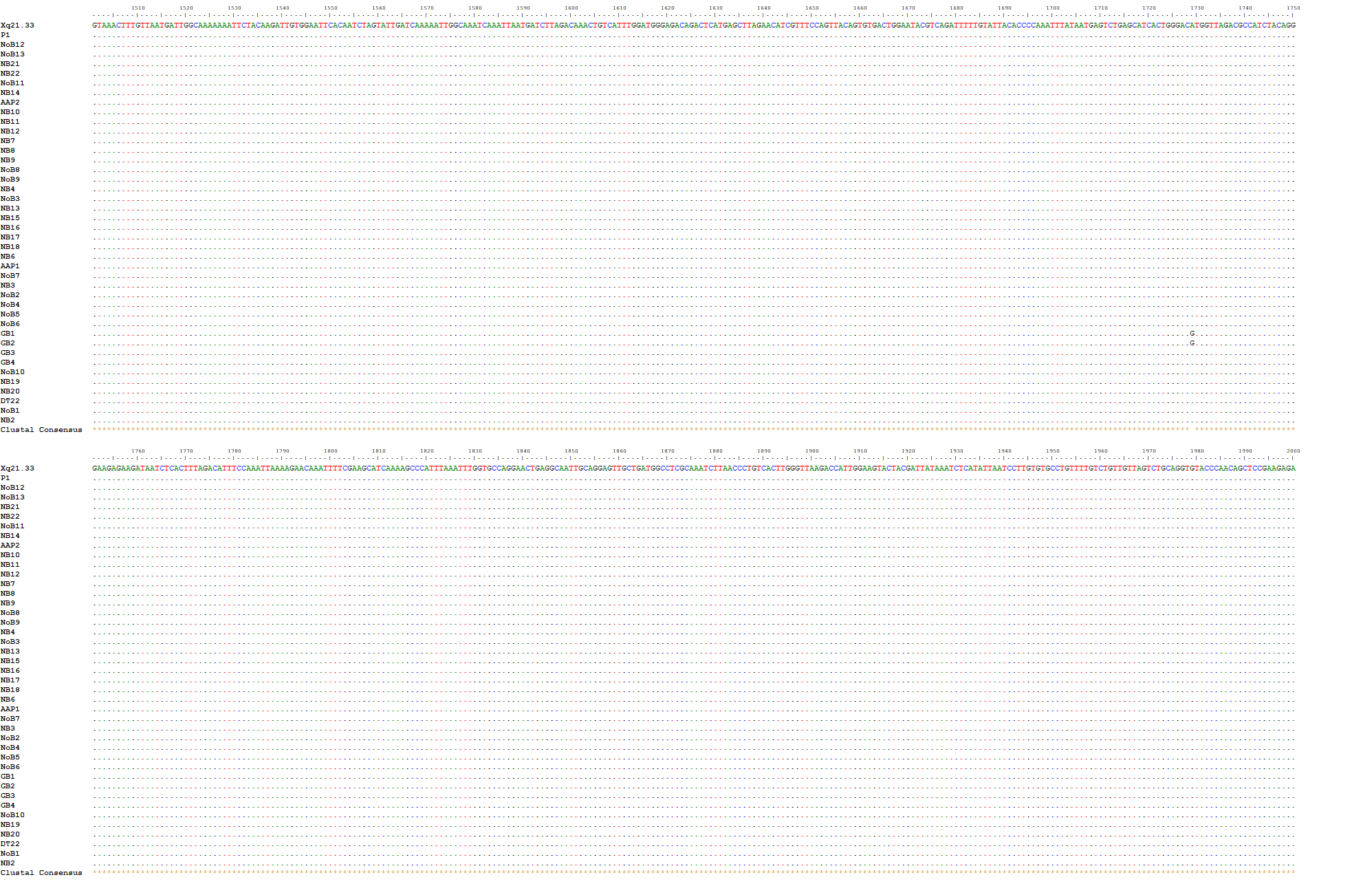


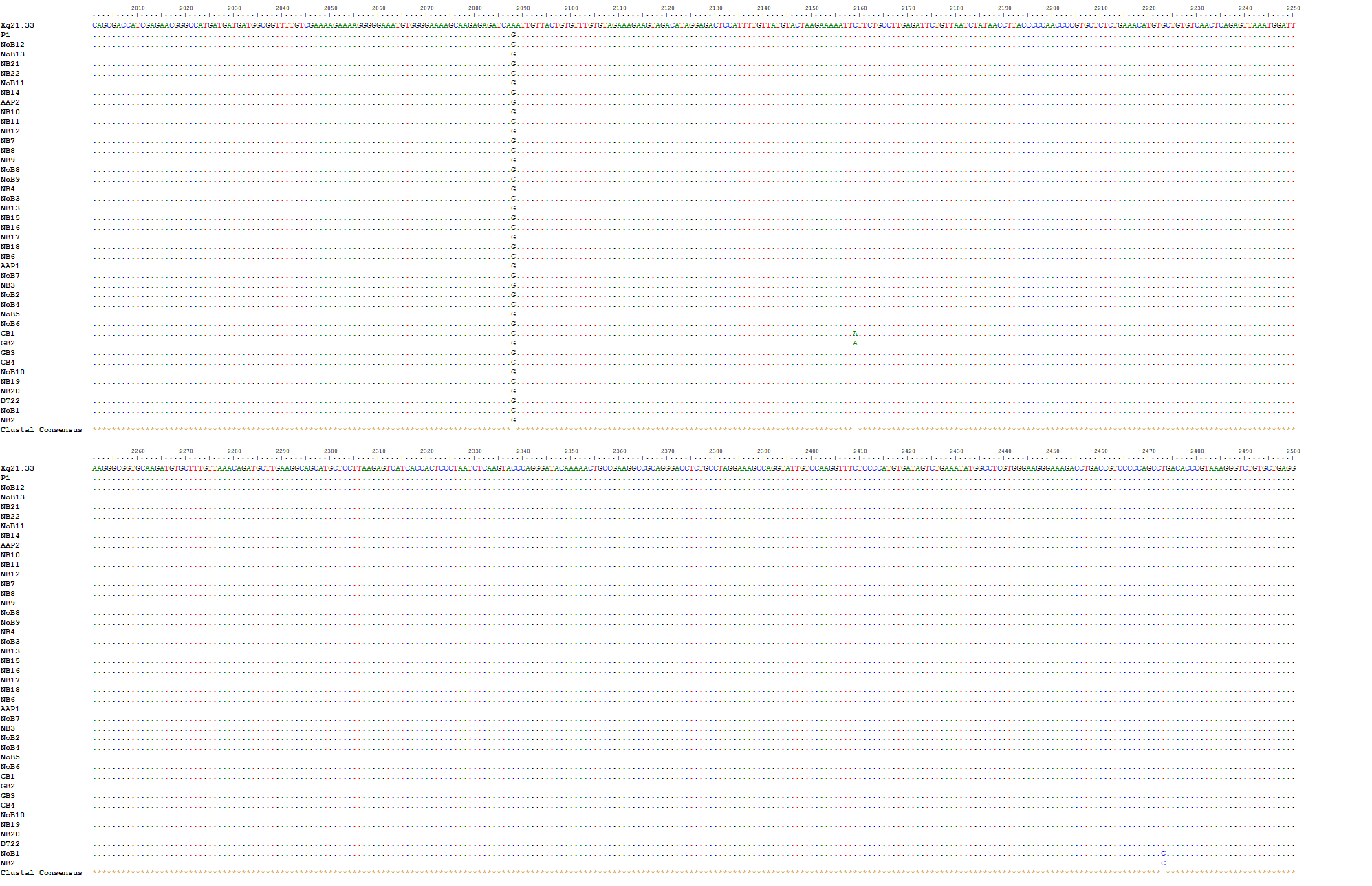


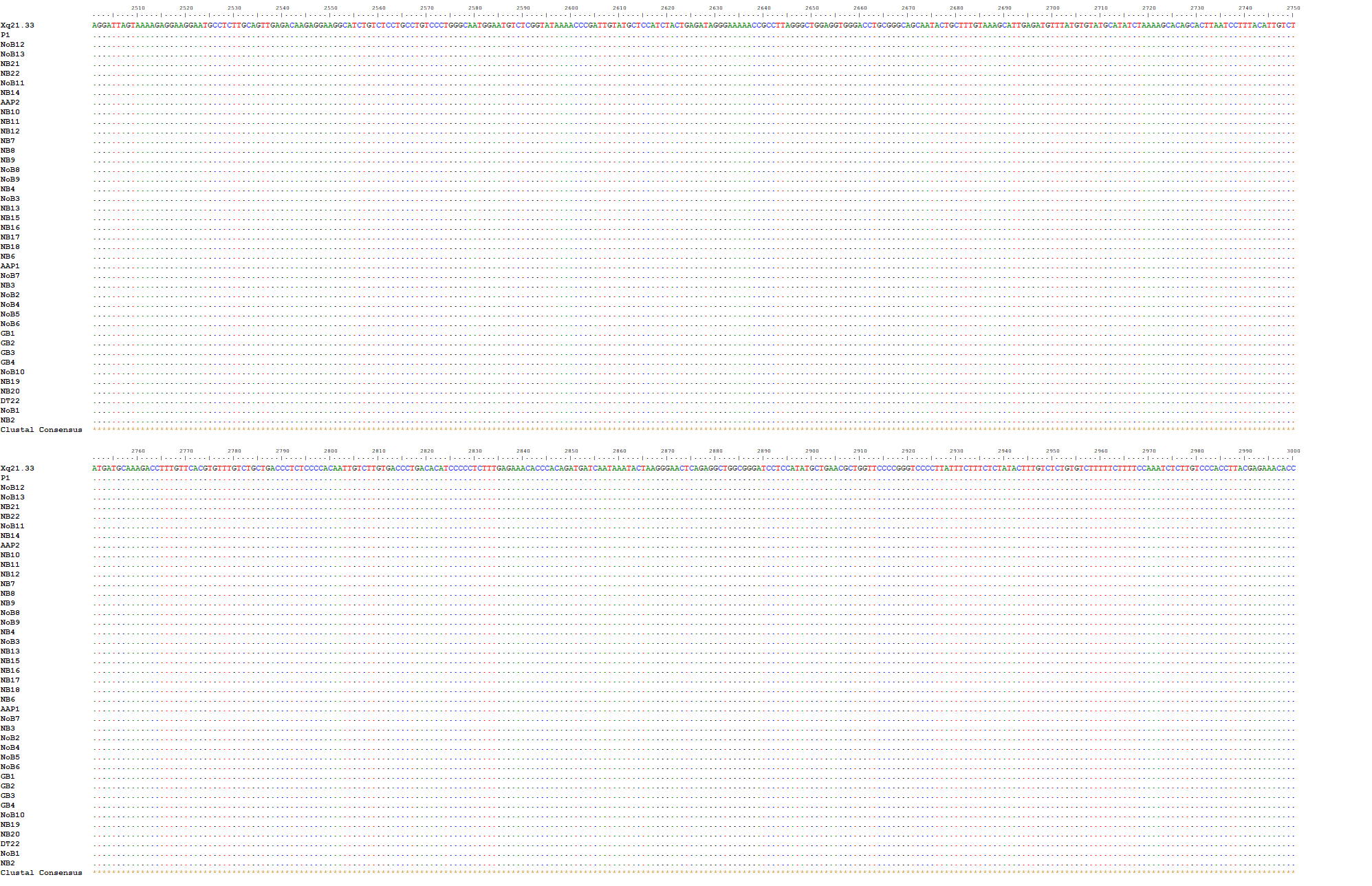


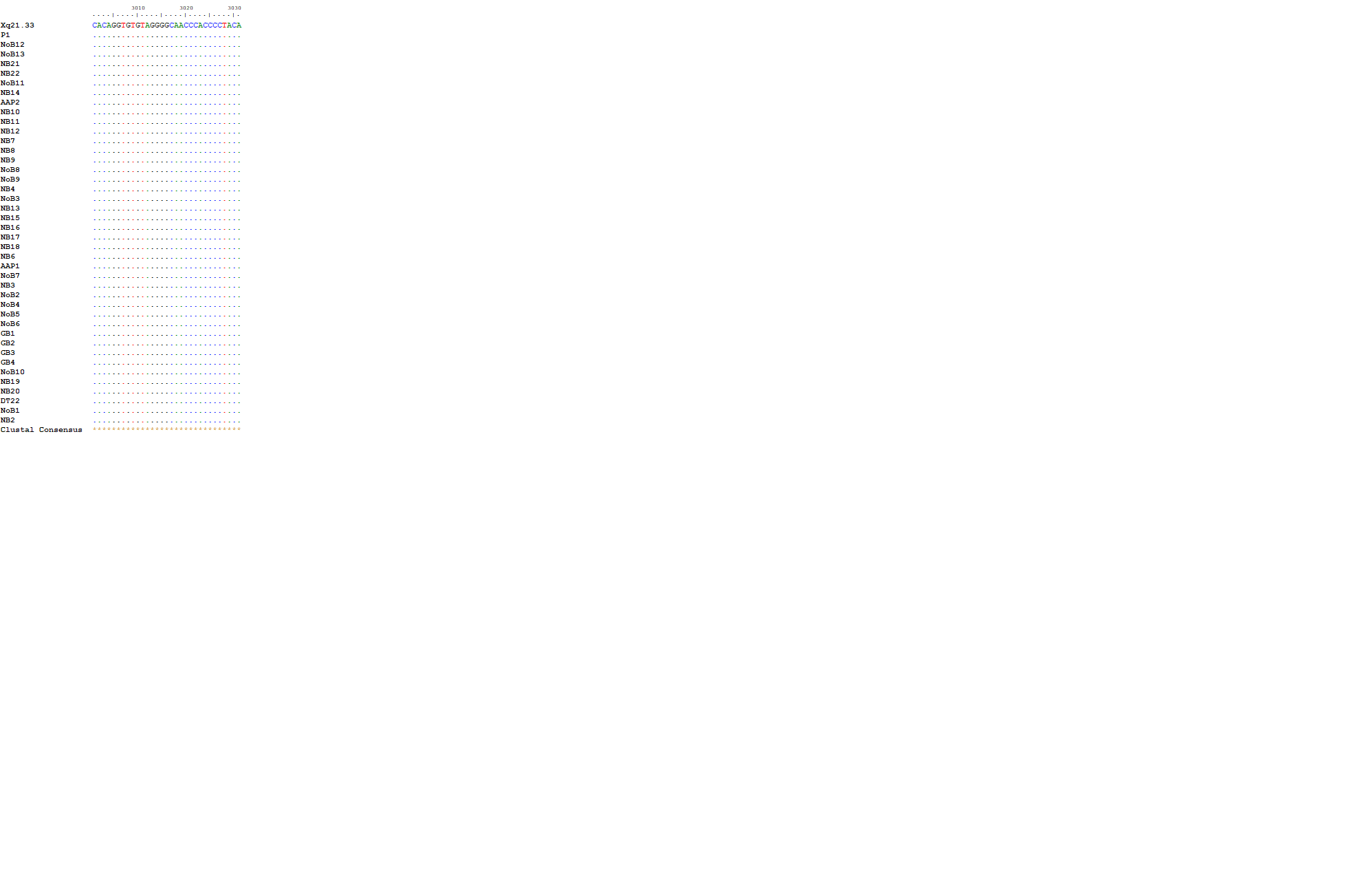

Supplement: Supplementary file 1 — Additional file 1: Supplementary Fig. 1. Alignment of HK2 X21q33 sequences from populations of African ancestry. [file 13027_2020_284_MOESM1_ESM.docx]
